# First detection of *Ixodiphagus hookeri* (Hymenoptera: Encyrtidae) in *Ixodes ricinus* ticks (Acari: Ixodidae) from multiple locations in Hungary

**DOI:** 10.1038/s41598-023-28969-3

**Published:** 2023-01-28

**Authors:** Adrienn Gréta Tóth, Róbert Farkas, Mónika Gyurkovszky, Eszter Krikó, Norbert Solymosi

**Affiliations:** 1 Centre for Bioinformatics, University of Veterinary Medicine, Budapest, István u. 2., 1078 Hungary; 2 Department of Parasitology and Zoology, University of Veterinary Medicine, Budapest, István u. 2., 1078 Hungary

**Keywords:** Classification and taxonomy, Invasive species, Entomology

## Abstract

The parasitoid wasp, *Ixodiphagus hookeri* (Hymenoptera: Encyrtidae), is the natural enemy of a wide range of hard and soft tick species. While these encyrtid wasps are supposed to be distributed worldwide, only a few studies report on their actual distribution around the globe. Within a shotgun sequencing-based metagenome analysis, the occurrence of *I. hookeri* was screened at multiple *Ixodes ricinus* (Acari: Ixodidae) tick sampling points in Hungary to contribute to the assessment of the distribution patterns of the parasitoid wasps in Central Europe. To our knowledge, the first report of the species in Hungary and the description of the southernmost *I. hookeri* associated geoposition in Central Europe took place within our study. *I. hookeri* infested *I. ricinus* nymphs were detected at five sampling points in Hungary. The results show that the exact distribution range of *I. hookeri* is still barely studied. At the same time, unprecedented public health issues being brought about by climate change might require steps toward the exploitation of the tick biocontrol potential and as an ecological bioindicator role of the parasitoid wasp in the future.

## Introduction

The emergence of vector-borne diseases that can affect human and animal populations is strongly influenced by climate change, urbanization, and globalization^1^. Among the most significant arthropod vectors (e.g. ticks, fleas, black flies, mosquitoes, or sand flies), ticks transmit a markedly broad spectrum of pathogenic microorganisms, including various protozoa, rickettsiae, spirochaetes, and viruses^2^. Since Hungary is situated in the southern part of Central Europe, climate change may facilitate the expansion of certain tick species to this landlocked country from the neighboring Mediterranean region. Currently, 27 hard tick species (Ixodidae) have been described in Hungary^3^. As part of the VectorNet project, the European Centre of Disease Prevention and Control (ECDC) currently monitors seven tick species, including *Ixodes ricinus*, that commonly transfer diseases to humans and animals^4^. Naturally, further tick species may also serve as vectors for microorganisms of human or veterinary medical significance^5^. Out of the seven tick species monitored by ECDC, all except for *Ornithodoros* spp. are hard ticks present in Hungary^3^.

Several studies report the incidence trends of tick-borne encephalitis (TBE) and Lyme borreliosis (LB), the most prevalent tick-borne infection in Europe^6,7^. In the case of LB, which has higher country-wise incidence rates than TBE, decade-long trends of incidence rates are not consistent along the countries around the world, increasing, and decreasing tendencies both appear. On the other hand, reports of the geographic distribution of LB show a clear expansion, especially towards higher altitudes and latitudes^6,7^. Although global trends of the incidence rates of these TBDs are not consistent^6,7^, changes in the distribution range of European *I. ricinus* populations are. Enhanced surveillance and diagnostic measures raise awareness of the changing geographical distribution, density, and activity of the *I. ricinus*, the primary vector of TBE and LB in Europe^8^. As a consequence of climate change, *I. ricinus* expanded its distribution to areas of higher altitude and latitude apart from its prior range, and its northerly shift within the European continent has also been documented^9–11^.

As climate change, accompanied by various sociodemographic alterations, brings unprecedented challenges related to vector-borne diseases^12,13^, the need for the development of control methods against tick populations is a public highlight. Several methods have been introduced to address this issue. These control methods often rest on either conventional chemical acaricides or on further alternatives, such as biological control methods assisted by the natural enemies of ticks^14–18^. A line of biological control methods against ticks could be the Encyrtidae family members which are small-sized, parasitoid, or hyperparasitoid wasps distributed all around the globe. Due to their efficacy and target specificity, numerous wasps from this family are used as biological pest control, while several additional encyrtid species are documented as promising candidates for this role^19–21^. *Ixodiphagus* spp., including *I. hookeri*^22^, are encyrtid wasps attacking a wide range of tick species that have received relatively much attention as a specific and effective, natural alternative for biological hard tick control^23,24^. Interestingly, *I. hookeri* appears to have alternating preferences for the tick species and developmental stage of its hosts at geographically distant locations^25,26^. In European settings, *Ixodiphagus* wasps are described to parasitize the larvae and nymphs of hard ticks with a clear predilection for unfed nymphs^24^. If oviposition occurs in larvae, transstadial transmission through the molting of the ticks to nymphs can also occur^27^. Wasp eggs start their embryonic development in engorging or engorged nymphs. Wasp larvae feed on tick tissues and emerge as fully grown adults causing the death of the host before it can reach the adult stage^23,24^.

*Ixodiphagus* wasps have been associated with several hard and soft tick genera belonging to the families of Ixodidae and Argasidae, including *Ornithodoros*, *Amblyomma*, *Dermacentor*, *Haemaphysalis*, *Hyalomma*, *Ixodes* and *Rhipicephalus*^23^. Studies conducted in Europe revealed that *I. ricinus* appears to be the preferred species of the European *I. hookeri*, while another common tick species, *Dermacentor reticulatus* is supposed not to be chosen as a host by the European representatives of the parasitoid wasps^24^.

While *Ixodiphagus* spp. have been detected in many countries and in a diverse range of hard and soft tick species, parasitoid wasps have been less studied in Hungary despite their potential to reduce tick populations and tick-borne disease cases. In the present study, our aim was to confirm the presence *I. hookeri* in a diverse set of locations in Hungary using a modern, sensitive metagenomic approach^28,29^. Due to their high density in Hungary, high public health significance as TBD transmitters, and potential to host *Ixodiphagus* wasps, *I. ricinus* ticks were decided to be assessed for the parasitoids. Based on our approach, genomic information of the European populations of *I. hookeri* may also be obtained, which can serve as a reference for further studies.

## Materials and methods

Between March and August of 2019, in two *I. ricinus* metagenome surveys, questing ticks, were collected from 21 geopositions in Hungary by flagging and dragging. One of the surveys was performed as a country-wide climatically designed sampling (17 sites)^30^. Further samples were collected in 4 sites popular for outings and dog walking. The closest settlements of sampling points were: Kissziget (a), Sárvár (b), Mosonmagyaróvár (c), Sáska (d), Darány (e), Somogybabod (f), Pénzesgyőr (g), Pécsvárad (h), Vérteskozma (i), Németkér (j), Törökbálint (k), Normafa (l), Nagy-Hideg-Hegy (m), 10th district of Budapest (n), Pusztavacs (o), Kékes (p), Lillafüred (q), Aggtelek (r), Háromhuta (s), Nyíregyháza (t), Nyíradony (u). The collected ticks were frozen at − 18 °C. In the laboratory, the ticks were classified taxonomically using standard morphological keys^31^, and 10 nymphs and 10 adult females of *I. ricinus* per sampling sites (Fig. 2) were selected randomly. Before DNA extraction, the ticks were washed twice with 99.8% alcohol.

The blackPREP Tick DNA/RNA Kit (Analytik Jena GmbH) was used for the DNA isolation. Isolated total metagenome DNA was used for library preparation. In vitro fragment libraries were prepared using the NEBNext Ultra II DNA Library Prep Kit for Illumina. Paired-end fragment reads were generated on an Illumina NextSeq sequencer using TG NextSeq 500/550 High Output Kit v2 (300 cycles). Primary data analysis (base-calling) was carried out with Bbcl2fastq software (v2.17.1.14, Illumina).

On the raw sequencing data, quality-based filtering and trimming were performed by TrimGalore (v.0.6.6, https://github.com/FelixKrueger/TrimGalore), setting 20 as a quality threshold, retaining reads longer than 50 bp only. Using the remained reads, a de novo assembly was performed by MEGAHIT (v1.2.9)^32^ using default settings. The resulting contigs were taxonomically classified using Kraken2 (*k* = 35)^33^ with the NCBI non-redundant nucleotide database^34^. Contigs were predicted as *I. hookeri* by taxon classification and were checked with BLAST^35^ on the partial sequence of *I. hookeri* 28S ribosomal RNA gene (MH077537.1) as a reference. Multiple sequence alignment was done by MAFFT (v7.490)^36^. All data management procedures, analyses, and plottings^37,38^ were performed in the R environment (v4.2.1)^39^.

## Results

**Figure 1:**
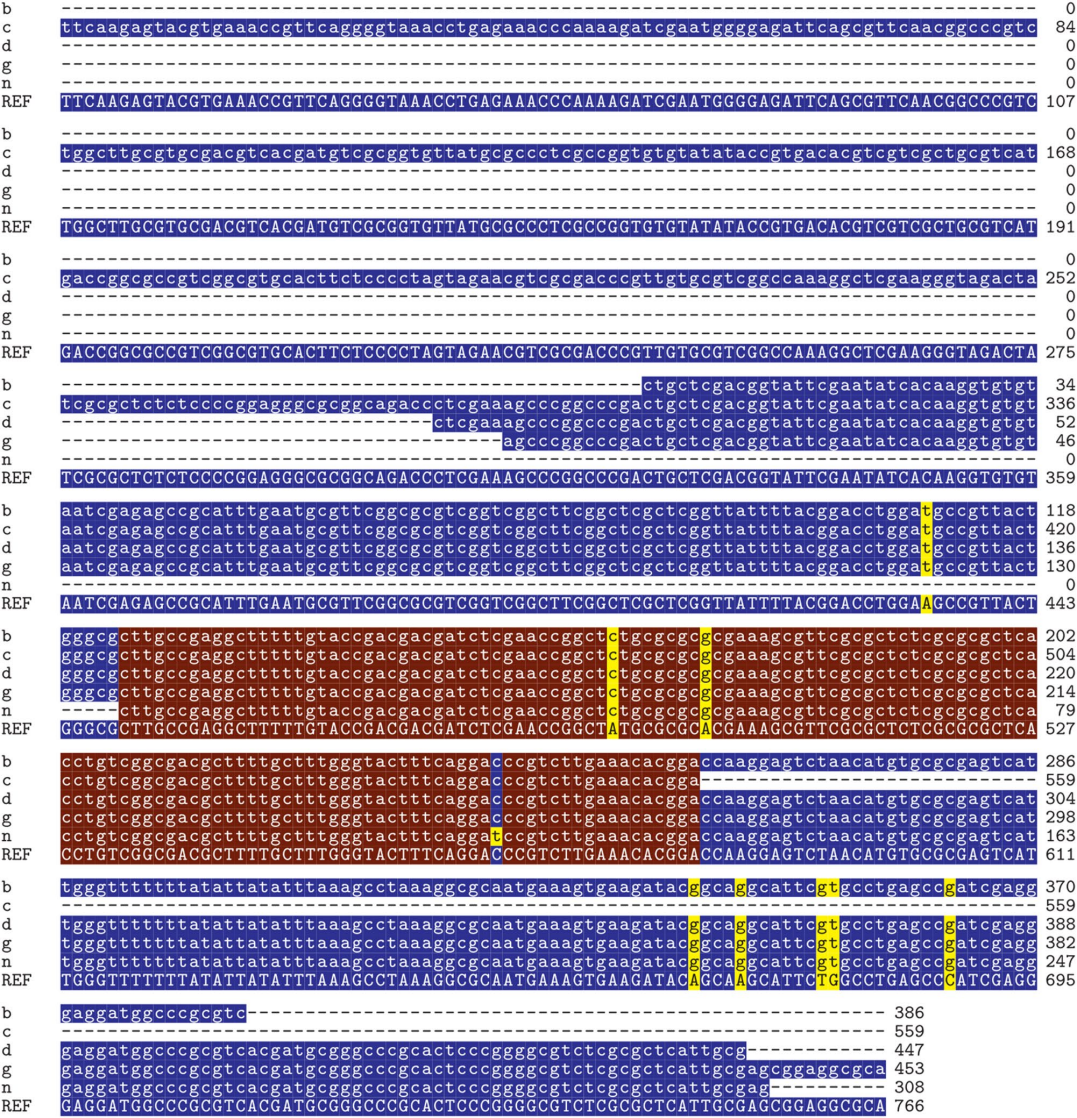
Multiple sequence alignments. The contigs were predicted to have *I. hookeri* origins based on the partial reference sequence of *I. hookeri* 28S ribosomal RNA gene (MH077537.1). Letters on a blue background indicate sequence identity from less than all the samples, and letters on a brown background indicate the parts where all 5 samples and the reference sequence are identical. Positions with yellow backgrounds indicate polymorphisms. The geopositions of the Hungarian samples are presented in Fig. 2.

**Figure 2:**
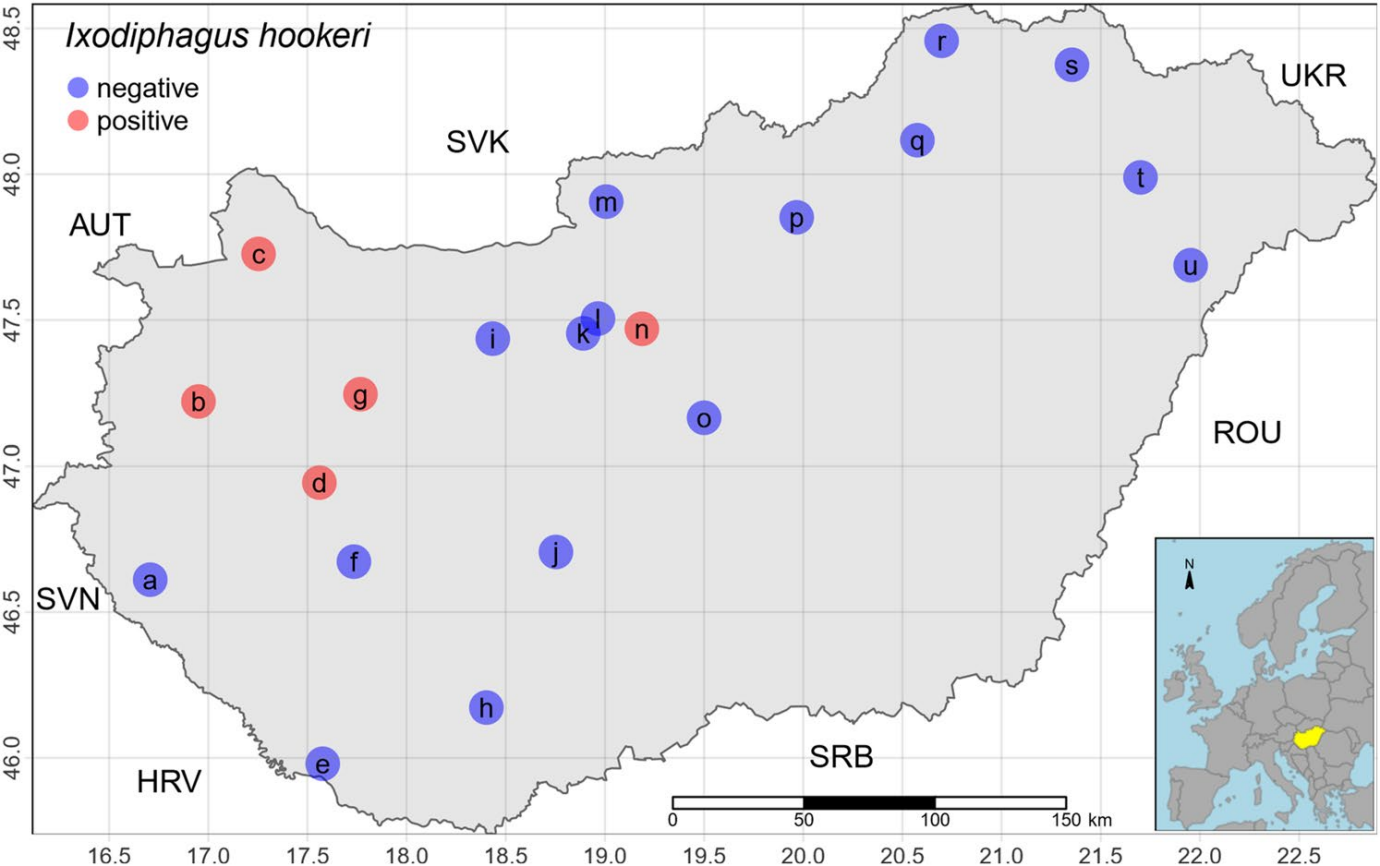
Geopositions of the metagenomically analyzed samples in Hungary. The red dots represent the sampling points we have found *I. hookeri* sequences, the blue ones where we have not. The inset map shows the study region, Hungary in Europe, colored yellow. Neighboring countries are presented by ISO 3 character codes (AUT: Austria, HRV: Croatia, ROU: Romania, SRB: Serbia, SVK: Slovakia, SVN: Slovenia, UKR: Ukraine).

Of the 21 adult female samples (10 individuals per sample) examined, we did not find any contigs with reasonable evidence for *I. hookeri* origin. In five of the 21 nymph samples (10 individual nymphs per sample), namely sample b, c, d, g, n, contigs deriving from I*. hookeri* were found. The sequence identity of the contigs deposited in GenBank to the *I. hookeri* 28S rRNA gene was 378/386 (97.9%), 556/559 (99.4%), 439/447 (98.2%), 445/453 (98.2%), and 300/308 (97.4%) for samples b (accession id: OQ316579), c (OQ316577), d (OQ316581), g (OQ316578), and n (OQ312115), respectively. The multiple sequence alignments of the contigs with the partial reference sequence of *I. hookeri* 28S rRNA gene (MH077537.1) are shown in Fig. 1.The figure shows that the sequence of the generated contigs varies from the reference sequence at 8 positions (A434T, A491C, A499G, A666G, A670G, T677G, G678T, C688G). In the annotation of the altered positions, the first letter refers to the base in the reference sequence, the following numbers specify the genomic position of the polymorphism, and the last letter indicates the base detected in our samples. By position A434T, transversions were identified in sample b, c, d, and g. All our samples included A491C and A499G mutations. A666G, A670G, T677G, G678T, and C688G polymorphisms occurred in sample b, d, g, and n. In the easternmost sample (n), we found one position (C565T) that differs from all other Hungarian samples and the reference sequence as well. The geoposition of the samples is presented in Fig. 2.

## Discussion

The findings that no reads deriving from *I. hookeri* were detected in adult *I. ricinus* samples collected between the end of March and the middle of May, while nymphs were associated with this species, promote former theories of the life cycle of the parasitoid wasps^23,24^. *I. hookeri* eggs may have been laid in the larvae or in nymphs before winter or, less probably, during spring, as the wasps were formerly associated with the possibility of surviving winter conditions^40,41^.

To our knowledge, this is the first report on evidence of the presence of *Ixodiphagus* wasps, namely *I. hookeri* in Hungary. This finding expands the localities associated with *I. hookeri* within Europe. All except one sampling points that were proven to host *I. hookeri* are located in western Hungary. The cluster of the four western Hungarian sampling points lays close to Austria’s and Slovakia’s borders. While the presence of *I. hookeri* has not been published in Austria; to our best knowledge, Slovakian reports of the occurrence of the wasps exist. *I. hookeri* has previously been identified at three locations within Slovakia; near Šoporña, associated to *Haemaphysalis concinna*^42^, close to the capital of Slovakia, Bratislava, in *I. ricinus*^43,44^ and in the Slovak Karst, isolated from both *I. ricinus* and *H. concinna*^41^. According to our hypothesis, the western Hungarian cluster of sampling points b, c, d, and g (Sárvár, Mosonmagyaróvár, Sáska, and Pénzesgyőr, respectively) may be associated with˝ the wasp populations described by Bratislava^43,44^ and by Šoporña^42^ in Slovakia. Despite being situated close to areas where *I. hookeri* is present, no *I. hookeri* DNA was detected from ticks in sampling points m, p, q, r, and s (Nagy-Hideg-Hegy, Kékes, Lillafüred, Aggtelek, and Háromhuta, respectively). Shifting slightly to the east, sampling point n (Budapest) represented the closest occurrence of the wasps to Slovakia. Considering the physical proximity between sampling point r and the Slovak Karst, where the report of Buczek and colleagues was released, the possibility of receiving false negative results is raised. The basis of receiving false negative results will be described further on.

Other European countries where the presence of *I. hookeri* has been reported include the Czech Republic (detected in former Czechoslovakia)^45^, Finland^46^, France^47,48^, the Georgia^49^, Germany^24,26,50^, Italy^51^, the Netherlands^27,52^, the United Kingdom^53^, Russia (detected in the Ussuri forest, in the Asian part of the former Soviet Union)^54^ and Ukraine (detected in the former Soviet Union)^55^. To our knowledge, sampling point d (Sáska) in Hungary represents the southernmost detection point of *I. hookeri* within Central Europe. The detection of *I. hookeri* in Hungary may serve as a novel hint regarding the potential distribution *I. hookeri* at the Balkan Peninsula, where the species appears to be little studied.

As mentioned above, wasp-negative sampling points can be wasp-invaded. Even though next-generation sequencing (NGS) based metagenomic approach appears to be just as or even more sensitive as polymerase chain reaction (PCR) based target detection techniques^28,29,56^, certain limitations can be addressed. Within the pool of reads deriving from the shotgun-sequenced metagenome that contains genome fragments from every organism present in the sample, lower relative abundance rates of an individual species serve with relatively lower read counts from its genome. In other words, shotgun sequencing preserves the original relative read abundance rates of the various organisms of the samples and may represent fewer reads of certain species by non-targeted runs^57,58^. Moreover, the *I. hookeri* reference sequence, that the metagenomic read sets were aligned to only represented a smaller fragment, namely the unique 28S ribosomal RNA gene of the full *I. hookeri* genome. Thus only *I. hookeri* reads deriving from this part of the genome could have been aligned, that further increases the chance of false-negative sampling points for the wasps. Besides the above-mentioned reasons, due to the low European tick parasitization rates of *I. hookeri*, the 10 nymphs collected at the sampling points may miss the wasps by chance alone.

Nevertheless, NGS-based approaches have a prospering future within the studies of parasitoids of public health significance, such as *I. hookeri*. According to Collatz and colleagues, large geographical distance and climatic differences (e.g. presence in Africa, Asia, Europe, and North America)^24,25,59,60^ may even underlie divergence and distinct taxonomic categorization of *I. hookeri* to different species, subspecies or at least strains^24^. Concurrently, publications on *I. hookeri* indicate a certain extent of behavioral and host preferences at different continents^24,25,61^. To assess the basic variation in behavioral traits of *I. hookeri* or to identify specific characteristics of subgroups that can be better utilized by the biological control methods, the study of the *I. hookeri* genome or at least specific genome regions, such as 28S rRNA or 16S rRNA genes, may become inevitable, similarly to other weighty insect groups^52,62,63^.

The improvement of our knowledge of *I. hookeri* with either traditional or genomic methods could facilitate the assessment of its potential as a means of biological control, while limitations and doubts about the wasps’ biocontrol potential could be addressed with more research. Attempted mass releases of the parasitoid wasps in the U.S and in the former Soviet Union between 1920 and 1940^40,64,65^ were unsuccessful as far as causing noticeable reduction of tick populations. One reason for this may be that *I. hookeri* requires high tick host densities and superabundant tick populations to reach its ideal abundance^61,66^. Inadequate numbers of parasitoids released compared to the geographical areas may have also undermined these trials^23^. On the other hand, the parasitoid wasps have been transported to the sites of attempted mass releases from great distances, sometimes even from different continents (e.g. from France to the U.S.)^40,64^ without any considerations regarding their host preferences, climatic adaptations, or behavioral attributes, that have, since then turned out to be rather specific to their geographic locations of origin^24,25,61^. In a global perspective, climate-associated occurrence rate alterations^23,25^ or differences in other characteristics of *I. hookeri*, such as the duration of its development, may also be underlain by host-related factors. Synchronization with the maturation of the tick host and, indirectly, with the main activity period of the vertebrate hosts of the ticks throughout the year may play a role in the life cycle of the wasps^24^.

Furthermore, we do not know how great the tick populations would be without the endemic *I. hookeri* populations and how much the parasitoid wasps contribute to maintaining the equilibrium of the communities in which they are included^67^. Nonetheless, the hypothesis regarding sufficiently high tick host densities and superabundant parasitoid host populations is in line with findings regarding the bioindication potential of certain insect species, including parasitoid wasps^68^. If so, this potential may also be worth further observation.

Conclusively, assessment of existing populations and further examinations on entomologic and genomic traits along with ecological roles could help understand and exploit the *Ixodiphagus* wasps’ potential as a biological tick control method or as a potential bioindicator species.

## Data Availability

The datasets used and/or analyzed during the current study are available from the corresponding author upon reasonable request.
